# A procedure for localisation and electrophysiological characterisation of ion channels heterologously expressed in a plant context

**DOI:** 10.1186/1746-4811-1-14

**Published:** 2005-12-19

**Authors:** E Hosy, G Duby, A-A Véry, A Costa, H Sentenac, J-B Thibaud

**Affiliations:** 1 Biochimie et Physiologie Moléculaires des Plantes, UMR 5004, Agro-M/CNRS/INRA/UM2, F-34060 Montpellier Cedex 1, France; 2 Present address: Laboratoire de Biophysique Moléculaire et Cellulaire, UMR 5090, CEA-DRDC-BMC, 17 rue des Martyrs, F-38054 Grenoble Cedex 9, France; 3 Present address: Unité de Biochimie Physiologique, Institut des Sciences de la Vie, Université Catholique Louvain, Place Croix du Sud, 5-15, 1348 Louvain-la-Neuve, Belgium; 4 Present address: Division of Biology, Cell and Developmental Biology Section, and Center for Molecular Genetics, University of California San Diego, CA 92093-0116 La Jolla, USA

## Abstract

**Background:**

In silico analyses based on sequence similarities with animal channels have identified a large number of plant genes likely to encode ion channels. The attempts made to characterise such putative plant channels at the functional level have most often relied on electrophysiological analyses in classical expression systems, such as *Xenopus *oocytes or mammalian cells. In a number of cases, these expression systems have failed so far to provide functional data and one can speculate that using a plant expression system instead of an animal one might provide a more efficient way towards functional characterisation of plant channels, and a more realistic context to investigate regulation of plant channels.

**Results:**

With the aim of developing a plant expression system readily amenable to electrophysiological analyses, we optimised experimental conditions for preparation and transformation of tobacco mesophyll protoplasts and engineered expression plasmids, that were designed to allow subcellular localisation and functional characterisation of ion channels eventually in presence of their putative (possibly over-expressed) regulatory partners. Two inward K^+ ^channels from the Shaker family were functionally expressed in this system: not only the compliant KAT1 but also the recalcitrant AKT1 channel, which remains electrically silent when expressed in *Xenopus *oocytes or in mammalian cells.

**Conclusion:**

The level of endogenous currents in control protoplasts seems compatible with the use of the described experimental procedures for the characterisation of plant ion channels, by studying for instance their subcellular localisation, functional properties, structure-function relationships, interacting partners and regulation, very likely in a more realistic context than the classically used animal systems.

## Background

Ion channel activity is essential to living cells in both plants and animals. Classical (or "forward") electrophysiology consists in measuring the cell membrane conductance and in deciphering this complex parameter towards the identification of the underlying ion transport systems and subsequently in getting as much information as possible on their functional properties, pharmacology, regulation and integration in cellular functions. In the late 70's to early 80's, the invention of the patch-clamp technique allowed this to be performed down to the molecular "single channel" level. At about the same time, came another breakthrough: the cloning of genes encoding ion pumps and channels enabled the inception of what could be named "reverse" electrophysiology, *i*.*e*., taking a road that leads from the *ex-situ* characterisation of a given ion transport system to its function in the cell or organism. Since the early characterisation of an acetylcholine receptor and a Na^+ ^channel of electric fish [1,2], this route has increasingly been taken while the knowledge of genomes became available.

In plants, ion channels are acknowledged to play crucial roles in transport of nutrient ions and control of membrane potential, and to be players or final targets of signal transduction pathways [3]. *In planta*, electrophysiological analyses have revealed a wide array of ion channels, differing in their ionic selectivity (anion or cation channels, selective cation channels permeable to either K^+ ^or Ca^2+^, poorly selective channels, *etc.*), or in their activation mechanism (voltage-gated, stretch-activated, or ligand-gated channels). To date however, the molecular structures mediating many ionic currents described *in vivo *remain to be identified. Only a few plant channels, most of them belonging to the Shaker family [4], have been characterised at the molecular level and correlated with physiological functions. Little is known at the molecular level about non-selective cation channels, Ca^2+ ^channels, stretch-activated channels or anion channels.

A convenient and realistic heterologous expression system is a prerequisite for successful "reverse" electrophysiology investigations. An ideal system should consist in cells devoid of endogenous ion transport activity ("electrically silent") and widely permissive in terms of foreign gene expression. The most widely used systems, *Xenopus *oocytes [5] or cell lines such as COS, *Sf*9 or HEK cells [6-10] do not fulfil this key requirement, but they generally offer acceptable compromises. Plant electrophysiologists have however experienced that strategies relying on heterologous expression in animal contexts are often poorly efficient for characterisation of plant ion channels. For example, this has been true for some Shaker-like K^+ ^channels [11], cation channels from the putative cyclic nucleotide gated channel (CNGC) family [12], glutamate receptors [13,14] and anion channels (CLCs) [15]. Such difficulties might be due to problems in RNA translatability and protein targeting, or to differences in post-translational modifying processes or in control by interacting protein partners, between the plant native context and the animal heterologous one. Surprisingly, in some cases, after several failures to characterise a given plant channel in different animal expression systems, the use of another animal system can open the way to functional analyses. This has been the case for the *Arabidopsis *Shaker K^+ ^channel AKT1, which is electrically silent when expressed in *Xenopus *oocytes [11] but gives rise to hyperpolarisation activated inward currents when expressed in insect cells [16]. However, the search for the suitable system will remain quite empirical until the reasons why a given channel is expressed in a functional state or not in a given animal system are discovered.

Bei and Luan opened the way towards a "green" heterologous expression system when they found that tobacco mesophyll protoplasts are devoid of K^+ ^inward currents and demonstrated that the KAT1 Shaker channel could be heterologoulsy expressed and subsequently characterised therein [17]. The stable transformation protocol by Agrobacterium infiltration of leaf disks and subsequent regeneration of a plant [18], however, is time consuming. Another drawback of this method is that ubiquitous expression of some transgenes may, in some instances, prevent the regeneration of a transformed plant that is required to obtain mesophyll protoplasts, or induce inopportune transcriptome modifications. On the other hand, the transient expression of protein-GFP fusions in tobacco cells has been used since about 8 years to study the targeting of proteins [19,20] suggesting possible use of these cells for electrophysiological characterisation of electrogenic transport systems [21]. Based on this, we developed a new procedure relying on transient transformation of tobacco mesophyll protoplasts. Vectors (available upon request) were engineered that allow selection of the transformed protoplasts (GFP reporter), expression of GFP-tagged or untagged proteins (for subcellular localisation and electrophysiological analyses, respectively), and co-expression of two different proteins in order to investigate their functional interactions. A PEG-mediated transformation protocol was adapted and the potential usefulness of the method was assessed by functional expression of the AKT1 channel, a result that had not been obtained in *Xenopus *oocytes or COS cells as mentioned above. The whole set of data indicates that the method can indeed provide a new way to functional analyses of plant ion channels.

## Results

### Genetic constructions for transient expression in tobacco protoplasts

Expression plasmids were engineered to display the following key features: (i) tagging of transformed cells, (ii) obtaining high expression levels of the studied protein(s), (iii) allowing both subcellular localisation and functional characterisation of the protein(s) of interest.

Selection markers such as antibiotic resistance or GUS staining are not suited to selection of protoplasts intended for patch-clamping. We therefore opted for a GFP-based selection. Indeed, GFP is easily expressed in plant cells with few or no drawbacks, when not fused to another protein. Fusion of GFP to membrane proteins, K^+ ^channels in our case, can be a solution for both detecting transformed cells and studying tagged-polypeptides [21]. It cannot be excluded, however, that, in some instances, tag-fused polypeptides display altered function and/or impaired multimeric assembly or interaction with regulating factors [22]. Therefore, vectors were constructed to enable either expression of the protein of interest fused to GFP for subcellular localisation, or co-expression of this protein and of GFP for functional characterisation purposes, as detailed below.

**Figure 1:**
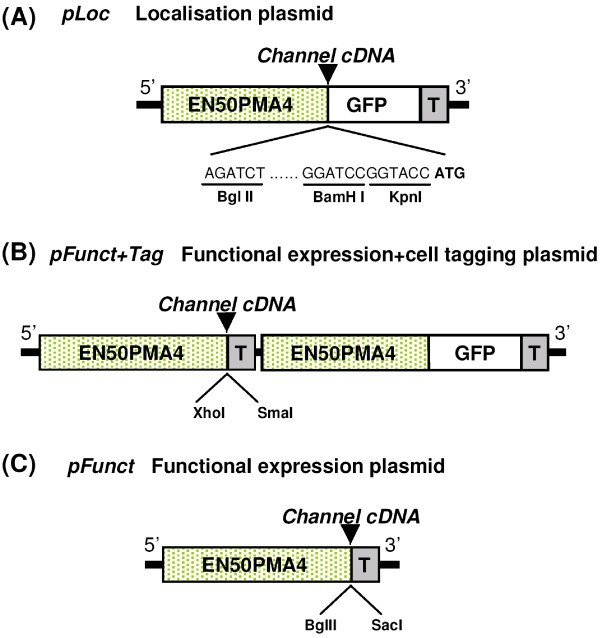
**Expression vectors engineered for transformation of tobacco mesophyll protoplasts**. (A) *pLoc*. This vector carries a single over-expression cassette. The green fluorescent protein cDNA (*"GFP"*) is framed by a strong promoter ("EN50PMA4") and a terminator ("T"). Three restriction sites (Bgl II, BamH I, Kpn I) are available for in-phase cloning of another cDNA. "ATG" indicates the first *GFP *codon. This vector allows the expression of fusion polypeptides with a GFP-tag in C-terminal for localisation purpose. (B) *pFunct+Tag*. This vector carries two expression cassettes both featuring the EN50PMA4 promoter and the T terminator. The first cassette encodes the GFP alone and the second one displays two restriction sites (Xho I, Sma I) for cDNA cloning. *pFunct+Tag *is used for functional expression of the recombinant cDNA while fluotagging transformed cells. (C) *pFunct*. This vector has the same construction as *pFunct+Tag *but the GFP cassette is absent. It is used, in combination of *pLoc *or *pFunct+Tag*, when two cDNAs are to be co-expressed (see text). EN50PMA4 is a tobacco enhanced promoter (see "Methods"); and T: is the nopaline synthase terminator of *Agrobacterium tumefaciens*. These vectors were obtained in a pTZ-19U plasmid (Stratagene, LaJolla, CA, USA) background.

Four plasmids containing one or two expression cassettes were constructed. Each expression cassette was featured with the *EN50PMA *promoter and the nopaline synthase terminator of *Agrobacterium tumefaciens *(noted *T *in Figure 1), a combination previously used for transient expression of mitochondria-targeted GFP in tobacco protoplasts [23]. The *EN50PMA *promoter consists of the *pma4 *(plasma membrane H^+ ^-ATPase isoform #4 gene) promoter in which the CaMV 35S enhancer is inserted at position -50 upstream from the transcription start of the *pma4 *gene. This promoter has been shown to yield expression levels at least ten fold higher than the classical CaMV 35S promoter [24].

In the first plasmid, called *pLoc*, a short polylinker (made of three restriction sites) was inserted between EN50PMA and the GFP-coding sequence (Figure 1A). This plasmid, expressing a GFP-tagged polypeptide with the polypeptide cDNA (free from STOP codon) cloned at the polylinker in frame with GFP, was used for subcellular localisation.

The second plasmid, called *pFunct+Tag *contained two expression cassettes, separately expressing the cloned polypeptide cDNA and GFP (Figure 1B). It was used for both functional expression of the polypeptide and fluo-tagging of transformed protoplasts.

The third plasmid, called *pFunct*, contained one single expression cassette allowing expression of the cloned polypeptide without a GFP-tag (Figure 1C). It can be used in co-transformation experiments. In such experiments, this vector, recombining one cDNA, can be co-expressed with either the *pLoc *or *pFunct+Tag *plasmids, recombining another cDNA, thereby allowing the study of interactions between the two encoded polypeptides, regarding their subcellular localisation or functional properties, respectively.

In control experiments, empty *pLoc *vector was used to express GFP alone. This was made for electrophysiological analysis of control protoplasts having undergone the transformation process without expression of a foreign cDNA (except *GFP*).

### Preparation and transformation of tobacco protoplasts

Tobacco leaf protoplasts have been obtained and subsequently transformed for GFP-enabled localisation purposes since about a decade [25,26]. We experienced, however, that obtaining protoplasts readily amenable to patch-clamp experiments required the digestion, purification and transformation steps to be optimised as described below (see the proposed protocol in "Methods"). The duration of digestion appeared to be critical for the success of patch-clamp experiments. Despite the fact that a high transformation efficiency was obtained on 15-hour digested cells, a 19-hour digestion time resulted in an increased gigaseal success rate, probably because of an improved accessibility of the patch pipette to the cell membrane. A high density solution (MLO6) was added to the final incubation medium (see "Methods") in order to increase the solution density and to separate, after a 7 minute-centrifugation at 110 g, the alive and floating protoplasts from the sedimented dead ones. Performing the digestion and the transformation in the dark and subsequently keeping the protoplasts in the dark at 19°C until patch-clamping, also facilitated gigaseals probably because darkness and moderate temperature slowed new cell wall synthesis.

The transformation was performed with a PEG-mediated technique. Several polymers of PEG were tested at different concentrations and the best yield of transformation (averaging 20%) was obtained with a 25% (w/v) concentration of PEG-6000. The electroporation transformation technique was also tested but a lot of cells did not withstand the electrical pulse and the yield of transformation was around 5%. Moreover, this technique requires a lot of DNA (30 μg) and cells (1.5 10^6 ^cells/transformation) [27] whereas, in our conditions, the PEG technique requires 5 μg DNA and around 5.10^5 ^cells only per transformation [28]. A critical step for transformation is purity of the DNA. In our experiments, Nucleobond^® ^AX affinity columns (Macherey-Nagel, Düren, Germany) were used for DNA purification and a drastic decrease in the efficiency of transformation was observed when old DNA preparations (> 1 month) or DNAs prepared by cell lysis and ethanol precipitation were used.

### Tobacco mesophyll protoplasts show weak inward K^+ ^and Cl^- ^currents

The native plasma membrane conductance of tobacco mesophyll protoplasts was investigated to test whether they could be used as hosts to express K^+ ^(or cationic) or/and Cl^- ^(anionic) channels. The patch-clamp technique was applied to non-transformed and to GFP-tagged (transformed with empty *pLoc *vector) protoplasts. In both cases, similar recordings were obtained revealing that the native membrane conductance was modified neither by the PEG treatment nor by expression of GFP, and that the PEG treatment did not alter the membrane amenability to patch-clamping (not shown).

**Figure 2:**
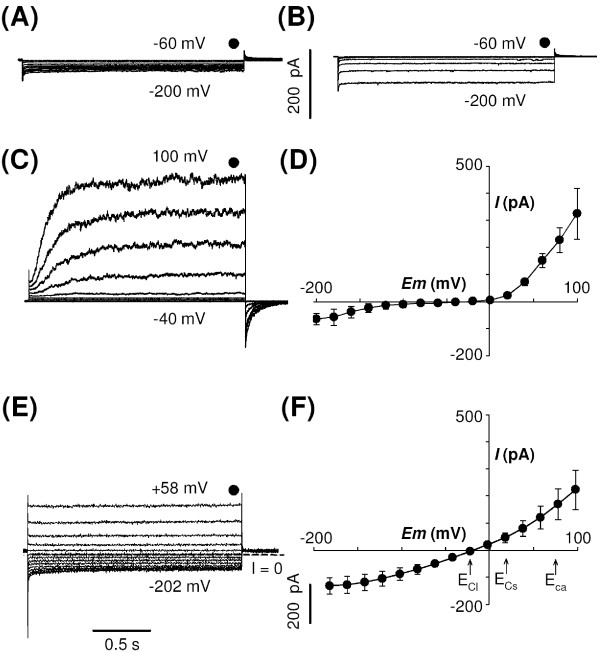
**Native K^+ ^and Cl^- ^currents in tobacco mesophyll protoplasts transformed by the empty *pLoc *vector**. (A, B) Typical recordings illustrating the two patterns of whole-cell inward K^+ ^currents elicited by membrane hyperpolarisation. 35 % of the patch-clamped protoplasts displayed the "no-current" pattern shown in (A). 65 % of the patch-clamped protoplasts displayed the voltage-dependent instantaneous weak current pattern shown in (B). (C) Typical recordings of whole-cell outward K^+ ^currents elicited by membrane depolarisation on the same protoplasts as in (A) and (B). The voltage steps ranged from -60 mV to -200 mV (A, B) and from -40 mV to +100 mV (C) in +20 mV increments, from a holding potential of -40 mV. The symbol above the records in a-c indicates the time of "steady-state" current sampling. (D) Average (mean ± SE, n = 10) of native steady-state K^+ ^currents in tobacco mesophyll protoplasts plotted against membrane potential. (E) Typical recordings of native whole-cell Cl^- ^currents recorded in protoplasts exposed to CsCl in pipette and extracellular solutions (see "Methods"). The voltage steps ranged from -202 mV to +58 mV in +20 mV increments and the holding potential was -22 mV. Dashed line marks zero current level. The symbol above the records indicates the time of "steady-state" current sampling. (F) Average (mean ± SE, n = 10) of native steady-state Cl^-^currents in tobacco mesophyll protoplasts plotted against membrane potential. Voltage dependence, at steady state, of the native chloride currents in tobacco mesophyll protoplasts (means ± SE; n = 10). E_Cl_, E_Cs _and E_Ca _represent equilibrium potentials for Cl^-^, Cs^+ ^and Ca^2+ ^respectively (see detailed content of bath and pipette solutions in "Methods").

Two patterns of native inwardly-rectifying K^+ ^currents could be observed during hyperpolarising pulses. About one third of the cells displayed virtually no inward current (Figure 2C). The other cells displayed low intensity (9 ± 3 pA.μm^-2 ^(n = 9) at -200 mV) currents, which activated instantaneously upon hyperpolarisation below -140 mV (Figure 2B), and which were insensitive to Cs^+^, a classical K^+ ^channel blocker (not shown).

Large (45 ± 13 pA.μm^-2 ^(n = 10) at +100 mV) outwardly-rectifying K^+ ^currents were activated by depolarising pulses (Figure 2C). These currents were probably due to the activity of Shaker channels as they displayed activation kinetics and extracellular potassium dependence reminiscent of those described for SKOR and GORK *Arabidopsis *outwardly-rectifying Shaker channels [29-31].

An average current-voltage relationship of control protoplasts (transformed with empty *pLoc *vector) is shown in Figure 2D. The low intensity of the instantaneous inward K^+ ^current seems compatible with the use of tobacco mesophyll protoplast as an expression system for heterologous inward K^+ ^channels.

Native anionic currents in these mesophyll protoplasts were recorded when Cl^- ^ions were the sole diffusible anions in both bath and pipette solutions (Cs^+ ^being added to block the K^+ ^currents). Outward currents were recorded (30 ± 9.6 pA.μm^-2 ^(n = 10) at +100 mV, Figure 2E–F), indicating that depolarisation-activated anion channels dominated the anionic conductance.

In summary, due to their moderate native conductances, tobacco mesophyll protoplasts seemed usable for characterisation of heterologously expressed K^+^(cation)- or Cl^-^(anion)-selective channels, preferably those channels mediating inward currents and possibly those mediating outward currents, provided that high heterologous expression level can be achieved.

### Functional expression and localisation of two inward K^+ ^channels

In the following, the use of tobacco protoplasts for transient expression of functional channels was evaluated with two model inwardly-rectifying Shaker channels cloned from *Arabidopsis*: AKT1 [32] and KAT1[33].

AKT1 had to be tested as it has only been functionally expressed in *Sf*9 (insect) cells [16] and yeast cells [32,34], but neither in *Xenopus *oocytes nor in COS cells so that this channel has been much less studied than KAT1. In contrast, the activity of KAT1 has successfully been recorded in a large set of heterologous expression systems: *Xenopus *oocytes [35,36], COS cells [37], *Sf*9 cells [38], yeast cells [34,39], tobacco mesophyll protoplasts [17] and *Vicia faba *guard cell protoplasts [21]. This strikingly compliant plant Shaker subunit has therefore been the most extensively described one, becoming the model plant Shaker channel for structure-function analysis. It was here used as a positive control of the usability of the transiently transformed tobacco protoplasts.

**Figure 3:**
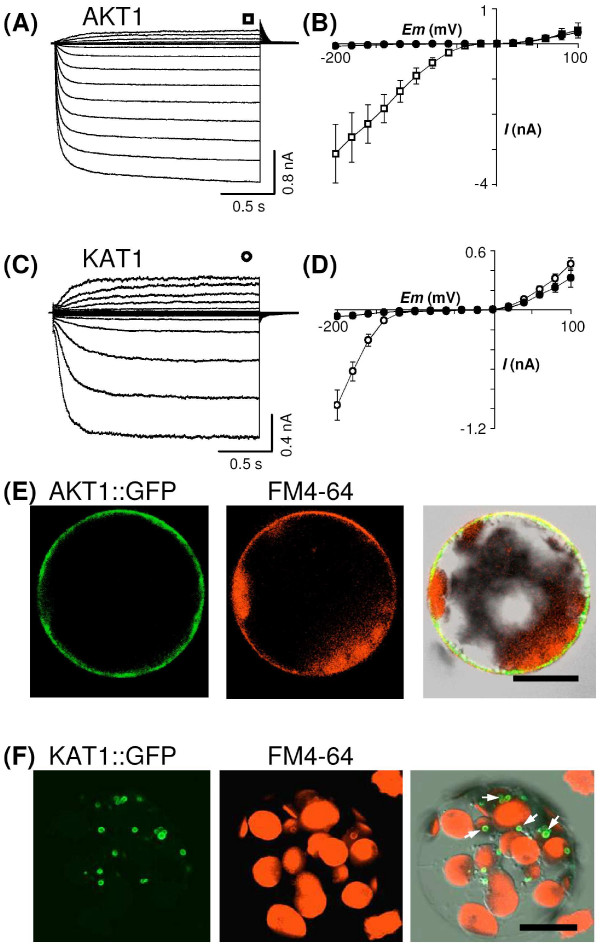
**Functional expression and subcellular localisation of AKT1 and KAT1 channels in tobacco mesophyll protoplasts**. (A) and (C) Typical recordings of the whole-cell inward and outward K^+ ^currents in patch-clamped tobacco mesophyll protoplasts respectively transformed with *AKT1*-carrying and *KAT1*-carrying *pFunct+Tag *vectors. The voltage steps ranged from -200 mV to +100 mV in 20 mV increments. The holding potential was 0 mV and -40 mV respectively for AKT1 and KAT1 expressing protoplasts. The symbol above the records in a and c indicates the time of "steady-state" current sampling. (B) and (D) Current-voltage relationships at steady state in control tobacco mesophyll protoplasts (closed circles in both B and D) and in AKT1-expressing (open squares in B) and KAT1-expressing (open circles in d) ones (means ± SE; n = 16 for AKT1, n = 13 for KAT1). (E, F) Confocal microscopy sections of protoplasts transformed with *AKT1*-carrying (E) and *KAT1*-carrying (F) *pLoc *vectors. The left panels display protoplast sections analysed for the GFP fluorescence, the middle panels the same sections analysed for chloroplast auto-fluorescence and FM4-64 fluorescence and the right panels the overlay of the two former panels with the transmission light image from the same protoplast section. FM4-64 was 50 μM in both (E) and (F) and was incubated for 10 min on ice in (E) and for 40 min at room temperature in (F). Some places where GFP and FM4-64 fluorescence co-localises are marked by white arrows in (F). Scale bar = 20 μm.

When patch-clamped, the GFP-labelled protoplasts transformed with the *pFunct+Tag-AKT1 *vector displayed exogenous voltage-gated K^+ ^currents, activating below a *ca*. -40 mV voltage threshold, exhibiting time dependent activation and inward rectification (Figure 3A) and reversing at a membrane potential close to the equilibrium potential for K^+ ^ions (not shown). These currents were similar to those observed in *Sf*9 cells infected by *AKT1*-recombinant bacculoviruses [16] or to the native ones recorded in root hairs of *atkc1-1 *knock-out plants, which are likely to express homomeric AKT1 channels [40]. An average (mean ± SE, n = 16) steady-state current/voltage curve for control and AKT1-expressing protoplasts is shown (Figure 3B) to highlight the marked change in inward currents, the outward currents being unchanged.

Electrophysiological recordings on *pFunct+Tag-KAT1*-transformed protoplasts revealed large exogenous inward-rectifying K^+ ^currents, which exhibited typical features of KAT1 currents [41], such as a time-dependent activation below a negative voltage threshold (here, *ca*. -120 mV; Figs. 3c and 3d).

The values of the voltage-gating parameters (activation threshold potential and, when available, so-called "Boltzmann" parameters, *i*.*e*. half-activation potential and apparent gating charge) previously obtained either in native or in heterologous contexts for AKT1 and KAT1 channels are compared to the ones obtained for these channels in the present work (Table 1). It appears that the values of these parameters depend on the expression context but that the present ones obtained in tobacco are closer to those obtained in the native context (*Arabidopsis*) than those obtained in animal or yeast cells.

**Table 1:** A comparison of the values of voltage-gating parameters of KAT1 and AKT1 channels in different expression contexts (see text).

**Channel**		**Mean value for voltage-gating parameters of the channel in**						**References**
			
		**Animal cells**			**Yeast cells**	**Plant cells**		
					
		**Xenopus oocytes**	**insect cells (*Sf*9)**	**Green monkey cos cells**		**Tobacco mesophyll cells**	**Arabidopsis native cells**	
**AKT1**	Activation potential (mV)	No current^(1)^	-70/-80 ^(1)^	No current^(2)^	-140^(3)^	-40	-40^(4)^	^(1) ^Ref [16]
	Gating charge	No current^(1)^	1.5^(1)^	No current^(2)^	n.r.^(3),*^	1.3 ± 0.2 (n = 5)	n.r. ^(4),*^	^(2) ^Erwan Michard, unpublished ^(3) ^Ref. [34]
	Half-activation potential (mV)	No current^(1)^	-123^(1)^	No current^(2)^	n.r.^(3),*^	-95 ± 10 (n = 5)	n.r. ^(4),*^	^(4) ^Ref. [40] ^(5) ^Ref. [35]
	
**KAT1**	Activation potential (mV)	-80^(5)^	- 60/-80^(6)^	- 100^(7)^	- 120^(3)^	-120	-120 ^(8)^	^(6) ^Ref. [38] ^(7) ^Ref. [37]
	Gating charge	1.7 ± 0.2 (n = 5)^(9)^	n.r.^(6),*^	1.6^(7)^	n.r.^(3),*^	1.7 ± 0.1 (n = 4)	1.6 ± 0.2 (n = 5)^(10)^	^(8) ^Ref. [46] ^(9) ^Ref. [36]
	Half-activation potential (mV)	-138 ± 7 (n = 5)^(9)^	n.r. ^(6),*^	-135^(7)^	n.r. ^(3),*^	-164 ± 6 (n = 4)	-157 ± 7 (n = 5)^(10)^	^(10) ^Eric Hosy, unpublished * not reported by these authors

**Figure 4:**
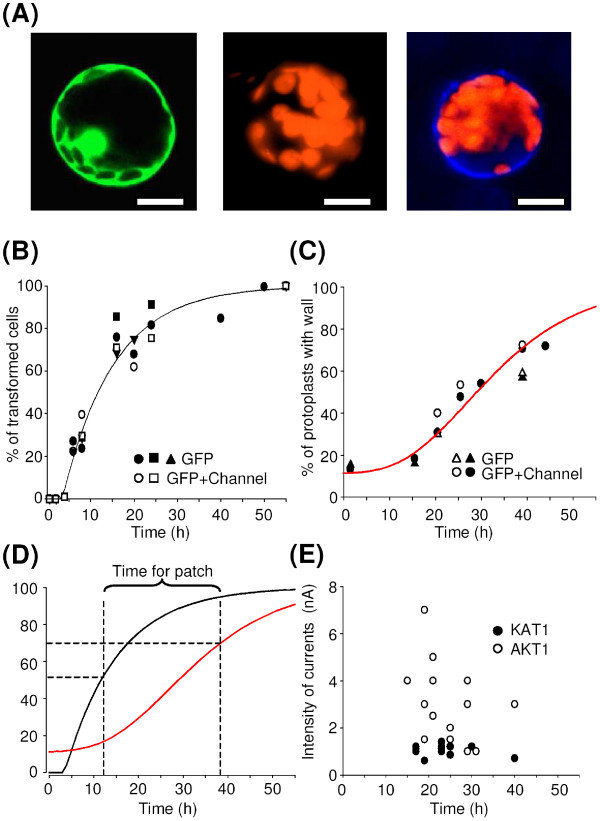
**Windowing the time allowed for patch-clamp recordings**. (A) Confocal microscopy sections of tobacco mesophyll protoplasts transformed with empty "localisation" plasmids (control protoplasts). Left panel: protoplast analysed for GFP detection. Middle and right panels: images of protoplasts bathing in calcofluor dye solution respectively without and with wall. Bar = 20 μm. (B) Time-course of GFP expression in transformed tobacco mesophyll protoplasts. The number of cells displaying GFP expression at a given time after transformation is expressed as a percentage of the number of cells which finally (55 hours after transformation) expressed GFP. Closed symbols: 3 independent transformations with empty *pLoc *("GFP"). Open symbols: 2 independent transformations with *pFunct+Tag-KAT1 *("channel+GFP"). About 500 transformed cells were considered for each experiment. Line represents exponential fit of the data. (C) Time-course of cell wall regeneration. The cell wall was marked with calcofluor dye. A cell was considered to have a wall if part of its surface showed blue staining. Each point represents about 200 protoplasts. Triangles and circles represent protoplasts transformed respectively with empty *pLoc *and *pFunct+Tag-KAT1*. Dark symbols represent all the protoplasts and open symbols those displaying green fluorescence. Line represents sigmoidal fit of the data. (D) Operational time window for patch-clamp recordings. Superimposition of the GFP apparition and cell wall synthesis fitted curves allows determination of a time frame for patch-clamp experiments (see text). (E) Time-course of the amplitude of steady-state currents recorded at -200 mV in tobacco mesophyll protoplasts transformed with *pFunct+Tag-KAT1 *(dark symbols) or *pFunct+Tag-AKT1 *(open symbols).

Protoplasts transformed with the *pLoc-AKT1 *vector displayed a green fluorescence distribution (Figure 3E, left photograph) suggesting targeting of the AKT1::GFP fusion protein to the cell plasma membrane. This was assessed by the green fluorescence co-localisation (Figure 3E, right photograph) with the red fluorescence of the membrane-marker FM4-64 (10 min incubation, Figure 3E, middle photograph). This pattern contrasted with the one displayed by protoplasts expressing GFP alone (not shown here but see Figure 4A, below). The membrane localisation of the GFP fluorescence in *pLoc-AKT1 *transformed protoplasts was fairly stable.

The situation was quite different with *pLoc-KAT1 *transformed protoplasts: the KAT1-attached GFP fluorescence was found essentially localised to 1 to 4 μm diameter structures (Figure 3F, left). These structures were assumed to be endocytic vesicles [42]. In order to check this hypothesis, the FM4-64 dye was incubated for 40 min with the protoplasts (Figure 3F, middle), a time allowing endocytic vesicles that appear upon vesicular internalisation of the plasma membrane to be labelled [43]. Many KAT1::GFP-labelled structures also displayed the red FM4-64 fluorescence (Figure 3F, right), suggesting that KAT1::GFP had undergone endocytosis. Similar intracellular GFP-labelled structures were already observed in KAT1::GFP-expressing *Vicia *guard cells and were also suggested to belong to the endocytic pathway [21].

### Co-expression of two polypeptides

The possibility of obtaining co-expression of two distinct polypeptides located on two different plasmids, as reported previously [28], was also tested using the present experimental procedures. PEG-mediated transformation of tobacco mesophyll protoplasts was attempted with a mixture of an empty *pLoc *vector and either *pFunct-AKT1 *or *pFunct-KAT1*. After this step, those cells having integrated the latter vector were spotted by green fluorescence. When patch-clamped, all the GFP-tagged protoplasts (of 25 AKT1 co-transformed protoplasts or of 23 KAT1 co-transformed ones) displayed exogenous currents (of AKT1- and KAT1-type, respectively). This evidenced that both vectors present in the transformation mixture were expressed in 100% of the transformed protoplasts.

### Transformed protoplasts can be used for at least 24 hours in patch-clamp experiments

To determine the best conditions for patch-clamp recordings in transformed tobacco protoplasts, the kinetics of green fluorescence apparition (reporting the expression of the exogenous proteins) and the kinetics of the wall reconstruction (limiting the access to the membrane for the pipette) were studied.

In a preliminary step, it was verified that every cell harbouring GFP expression that was patch-clamped displayed an exogenous conductance due to the activity of the studied channel (KAT1 and AKT1 channels being used as controls for functional expression of heterologous polypeptides). Green fluorescence (Figure 4A, left) was detectable as soon as 4 hours after the transformation step and the number of GFP-labelled cells subsequently reached a plateau by 55 hours following the transformation event (Figure 4B). Once this steady-state was reached, the yield of transformation could be estimated: it averaged 20 %. Interestingly, with respect to this final ratio of GFP-tagged cells, the time-course of the proportion of cells showing green fluorescence was fairly reproducible and poorly dependent on whether a channel was co-expressed or on which construct was used for this co-expression (Figure 4B). Operationally, one GFP-tagged cell amongst fifteen is an acceptable threshold for attempting patch-clamping on a cell culture dish. Hence, with a transformation efficiency of 20 %, the number of GFP-tagged cells on a dish was large enough to attempt patch-clamp recordings about 12 hours after the transformation process (Figure 4B).

In a second set of experiments, detection of cell wall regeneration by transformed protoplasts was monitored (Figure 4A, middle and right), using the calcofluor dye (Fluorescent brightener 28; Sigma). Except for the *ca*. 15 % of mis-digested protoplasts, which were stained immediately after the transformation, the first neo-synthesised cell wall pieces could be detected by 20 hours after the transformation (Figure 4C). Expression of GFP or both GFP and channel did not merely change the wall synthesis kinetics (Figure 4C). Sixty to 70 % of the protoplasts had synthesised (pieces of) a new wall by 40–45 hours after the transformation (Figure 4C), so that it became subsequently difficult to get good access to the membrane with the patch pipette.

These observations mean that the patch-clamp recordings can begin 12 hours after the transformation (expression time) and the currents can be measured up to 40 hours after the transformation. The time window for protoplasts usability is therefore at least 24 hours wide (Figure 4D). Interestingly, current intensity at -200 mV (here related to channel expression level) did not markedly increase with time after transformation (Figure 4E) and the expression level of channels in the membrane was high enough to record macroscopic currents as soon as GFP fluorescence was detectable.

In an attempt to extend the time allowed for patch-clamping, the newly synthesised cell wall was exposed to the previously used digest solution or to a solution enriched in enzymes. After 1, 2 or 5 hours of digestion, however, the quality of cell membrane obtained did not allow gigaseals. It could be hypothesised that isolated protoplasts generated walls with a composition different from that of leaf mesophyll cell walls, and that the enzyme cocktail could not permit a complete new digestion.

## Discussion

*In vitro *tobacco culture is straightforward and tobacco mesophyll cells can be obtained in large amounts so that the efficiency of the protoplast preparation is not limiting. The size of these cells is well adapted to the patch-clamp technique, and we observed that the synthesis of a new cell wall, which makes patch-clamping difficult can appreciably be slowed if cells are kept in the dark during digestion and after transformation. We show that, with the experimental procedure we have developed, a high yield of successful transformation can be routinely obtained and that the time frame for patch-clamp investigations is at least 20 hours wide (Figs. 1 and 4).

Endogenous activity of channels mediating K^+ ^inward currents is low in the PEG-transformed tobacco mesophyll protoplasts (Figure 2). This property is favourable for functional characterisation of inward K^+ ^channels expressed in this system. Indeed, our data demonstrate the functional expression in these protoplasts of AKT1, a channel so far refractory to expression in classical heterologous expression systems. AKT1 had been previously characterised only in the baculovirus-insect cell system [16] and in yeast cells [34]. Up to now, little has been reported on the functional properties of this channel, one of the first plant Shaker K^+ ^channels to be cloned [32], essentially because patch-clamping yeast is difficult and functional studies in *Sf*9 cells require previous purification of recombinant Baculoviruses, a highly time-consuming step, making this system poorly suitable for investigations of structure-function relationships using a number of mutant channels. The present report opens the way towards such investigations, as the expression vectors described here allow easy cDNA cloning and transformation.

A large number of plant genes sharing sequence similarities with animal genes known to encode ion channel subunits have been identified, for instance by *in silico *analysis of the *Arabidopsis *genome . Most of them are still uncharacterised at the functional level despite attempts to do this in classical animal expression systems. This is the case, for example, of the plant "glutamate receptor" genes [14] and "CNGCs" ones (putative cyclic nucleotide gated cation channels [12]), two gene families having been proposed to encode cation channels [4], and of the "CLC" genes, likely to encode anion channels [15]. In this context, it can be speculated that ion channels of other types than K^+ ^inward rectifiers could be characterised in tobacco mesophyll protoplasts. Channels mediating outward K^+ ^currents (Figure 2C) or channels mediating Ca^2+^(Ba^2+^) inward currents and activated by blue light [44] or cAMP [45] have been described in *Arabidopsis *mesophyll cells and are thus likely to be natively expressed also in tobacco mesophyll cells. Even for such channels, high heterologous expression levels in transformed protoplasts might open the way to their functional analyses.

A drawback in the use of heterologous systems is the fact that the expression context can affect channel functional features, providing a distorted view of the channel function and regulation. For example, the values of the voltage-gating parameters for activation of AKT1 and KAT1 clearly depend on the expression context (Table 1). Regarding KAT1, compared to the value obtained for the native hyperpolarisation-activated K^+ ^channels of *Arabidopsis *guard cells [46], the closest value is the one obtained in the present work (Table 1). Assuming that KAT1 channels do underlie the native *Arabidopsis *guard cell currents, this suggests that tobacco mesophyll protoplasts represent a more realistic expression context for plant ion channels than classically used animal cells.

Amongst the regulation mechanisms that could be studied using the tobacco mesophyll protoplast system are the events that enable channel targeting to the cell membrane and/or post translational modifications such as (de)phosphorylation. Concerning the former mechanisms, it is worth noting that KAT1 displayed the same subcellular localisation profile when expressed in tobacco mesophyll protoplasts as when expressed in *Vicia faba *guard cells [21].

Indeed, although peripheral localisation of (at least some) KAT1 channels is assessed by the exogenous currents recorded in protoplasts transformed by KAT1-carrying pFunct+Tag vectors (Figure 3B–D), KAT1::GFP is hardly seen at the membrane (Figure 3F) but rather appears, both in tobacco mesophyll protoplasts and *Vicia faba *guard cells, as mainly localised in punctate structures (Figure 3F), assumed to belong to the endocytosis pathway (Figure 3F, right photograph; [21]). Interestingly in AKT1::GFP-expressing protoplasts, the fluorescence was not seen in such punctate structures, but appeared as evenly distributed in the plasma membrane (Figure 3E). This indicates that KAT1 and AKT1 channels do not undergo the same regulation concerning their targeting to and/or withdrawal from the cell membrane. Thus, it can be proposed that regulation of K^+ ^Shaker channel activity involves specific control of the turn-over of the different Shaker subunits in the cell membrane.

A current challenge in ion channel research is the identification of regulatory proteins and the characterisation of the interacting networks they belong to. Progress in this field could be achieved by co-transformation of candidate partners in heterologous systems [47]. The possibility of obtaining co-expression of two distinct polypeptides located on two different plasmids is, therefore, a key feature of an appropriate heterologous expression system. This point had already been checked by immunostaining the nitrocellulose-transferred proteome from protoplasts expressing two different peptides [28]. Here, it has been assessed *in situ *within living cells and we conclude that PEG-mediated transformation of tobacco mesophyll protoplasts with a mixture of two vectors can be used to study the interaction of two polypeptides. These could be different channel subunits, or a channel subunit and a regulating partner, or any couple of putative interacting polypeptides. Depending on the vectors used, one could study the effect of the interaction on the cellular localisation or on the function of the polypeptides of interest.

## Conclusion

In conclusion, we show that transient expression in tobacco mesophyll protoplasts can provide a way to characterise plant ion channels, by studying for instance their subcellular localisation, functional properties, structure-function relationships, interacting partners and regulation, very likely in a more realistic context than the classically used animal systems. Clearly, the system is well adapted to characterisation of inward K^+ ^channels, and it can be expected to be usable also for the characterisation of other types of ion channels, such as poorly selective cation channels [48,49] or anion channels [15]. Furthermore, adaptation of the procedure to protoplasts prepared from *Arabidopsis *tissues, using plants of different genotypes, could provide even more straightforward routes to such analyses.

## Methods

### Plant material

*Nicotiana tabacum *(cv. SR1) plants were grown *in vitro *in a growth chamber at 22°C with a 16-h light/8-h dark regime with a photon flux density of 250 μmol.m^-2^.s^-1 ^on 0.8% agar medium containing MS/2 salts [50], 1 % sucrose and 5 mM MES-KOH pH 5.5).

### Genetic material

The vectors used for transient expression in tobacco mesophyll protoplasts contained either one expression cassette or two. Each cassette is fitted with the enhanced plant promoter of the plasma membrane H^+^-ATPase isoform 4 gene (pma4) from *Nicotiana plumbaginifolia *and the nopaline synthase terminator from *Agrobacterium tumefasciens *(see text and Figure 1A–C). The modified pTZ-19U (Stratagene, LaJolla, CA, USA) plasmid used for functional expression of Shaker channel cDNA in tobacco protoplasts contained two cassettes, allowing to co-express the GFP fluorescent marker protein and the Shaker cDNA sequence (*pFunct+Tag*, Figure 1B). XhoI and SmaI restriction sites were used to clone AKT1 and KAT1 cDNA into the expression cassette. The plasmid used for channel targeting contained only one expression cassette allowing the expression of a channel fused in its C-terminal part to the GFP protein (*pLoc*, Figure 1A). BglII restriction site and KpnI or BamHI sites were used to clone upstream the GFP, the AKT1 cDNA deprived of its STOP codon by PCR. Supplemental information on the vectors and on the cloning procedure used in this study is available as Additional file 1.

### Tobacco leaf mesophyll protoplast isolation

A previously described protocol [27] was adapted for obtaining purified leaf mesophyll protoplasts.

Five cm long tobacco leaves from 3- to 4-week-old plants grown in vitro were harvested under sterile conditions and their lower face was abrased with sandpaper n°1200. The leaves were then laid in Petri dishes containing 10 ml of the sterilised digestion EF medium (0.125% macerozyme R-10 (Yakult Pharmaceutical; Onozuka, Japan), 0.2% cellulase R-10 (Yakult Pharmaceutical; Onozuka, Japan), 5 mM CaCl_2_, 0.5 M sucrose, 0.1 % BSA, 2.5 MES-HCl, pH 5.2) for 19 h in the dark at 25°C. After digestion, undigested pieces of leaves were removed and 4 ml of floating MLO6 medium were added (15 mM CaCl_2_, 600 mM sucrose, 7.5 mM MES-KOH pH 6.0). The protoplast suspension was then filtered through a 100 μm nylon filter and centrifuged at 110 g for 7 min in a swinging rotor. The protoplasts localised in the floating band were harvested and diluted with 4 volumes of autoclaved washing W5 medium (154 mM NaCl, 125 mM CaCl_2_, 5 mM KCl, 5 mM glucose and 1.5 mM MES-KOH, pH 5.6). The cells were then pelleted (110 g for 7 min in a swinging rotor) and washed in 40 ml and then in 20 ml of "Mannitol/Mg" solution (15 mM MgCl_2_, 400 mM mannitol, 5 mM MES-KOH pH 5.6). Protoplasts were finally resuspended in Mannitol/Mg solution at a concentration of 10^6 ^cells/ml.

### Protoplast transformation

In an Eppendorf tube, 150 μl of the concentrated protoplasts and 150 μl of PEG solution (25% w/v PEG 6000, 0.45 M mannitol, 0.1 M Ca(NO_3_)_2_, pH 9.0) were added to 5 μg of plasmidic DNA from the construction to be tested and incubated at room temperature for 30 min. The sample was then diluted with 8 ml of K3M medium supplemented with 0.45 M glucose [25] and pelleted in a swinging rotor (7 min at 110 g). The protoplasts were finally suspended in 3 ml of K3M and incubated for 12 to 38 hours at 19°C in the dark before analysis.

### Subcellular localisation of channels fused to the GFP and detection of transformed protoplasts for electrophysiological measurements

Subcellular localisation of the channels fused to the GFP and of FM4-64 labelling in tobacco protoplasts was performed with a Zeiss confocal microscope (LSM510 AX70 Zeiss, Göttingen, Germany). For catching the GFP fluorescence, an excitation wavelength of 488 nm and a detection one between 500 and 530 nm were used. For catching the FM4-64 fluorescence, in KAT1::GFP expressing protoplasts, an excitation wavelength of 515 nm and a detection one above 640 nm were used, with a laser intensity at 60%. Different settings for FM4-64 detection were used in AKT1::GFP expressing protoplasts: excitation at 543 nm, detection above 585 nm and laser intensity at 15% (much lower chloroplast autofluorescence was catched with such settings, see Figure 3E). Images were treated with LSM510 sofware (Zeiss). Plasma membrane FM4-64 labelling was performed by incubating the protoplasts on ice 10 min in the culture medium supplemented with 50 μM FM4-64. For endocytic vesicle labelling, the incubation in 50 μM FM4-64 was prolonged up to 40 min and performed at room temperature. In order to measure currents only on transformed protoplasts, an epifluorescent microscope (IX 70, Olmpus, Hamburg, Germany) allowing GFP detection was combined with a patch-clamp set-up. Transformed cells were selected for patch-clamp analysis according to their GFP expression. GFP signal was detected between 489 nm and 508 nm using a emission filter (piston-GFP, Olympus) upon excitation at a wavelength of 488 nm emitted by a monochromator (Optoscan C80x, Cairn Research Ltd, UK). Images were acquired with a CCD camera (CoolSNAP HQ, Roper Scientific, USA) and treated with MetaFluor software (Universal Imaging Corporation, USA).

### Wall detection

To measure the kinetics of wall synthesis, the calcofluor white marquer (Fluorescent brightener 28, Sigma) was solubilised in 0.25 M Tris solution at the 0.005% w.v^-1 ^concentration. The calcofluor was excited with a 360–370 nm wavelenght and detected at above 420 nm with a high-pass emission filter (U-MNU2, Olympus) on an epifluorescent microscope (BX61, Olympus).

### Electrophysiological recording

Patch-clamp pipettes were pulled (P97, Sutter Instruments, Novato, CA) from borosilicate capillaries (Kimax-51, Kimble). Seals with resistances higher than 1 GΩ were used for electrophysiological analyses. Whole-cell recordings were obtained using an Axopatch 200A amplifier (Axon Instruments, Foster City, CA). pCLAMP 8 software (Axon Instruments) was used for voltage pulse stimulation, on-line data acquisition and data analysis. The voltage pulse protocols have been included in the figure legends.

### Electrophysiological solutions

For potassium current recordings, the pipette solution that equilibrates with the cytosol of mesophyll protoplasts contained 1 mM CaCl_2_, 5 mM EGTA, 2 mM MgCl_2_, 100 mM K-glutamate, 2 mM MgATP, 10 mM Hepes-NaOH, pH 7.5, osmolarity adjusted to 520 mOsM with D-mannitol. Mesophyll protoplasts were extracellulary perfused with a solution containing 10 mM CaCl_2_, 2 mM MgCl_2_, 50 mM K-glutamate, 10 mM MES-HCl, pH 5.5, osmolarity adjusted to 500 mOsM with D-mannitol. In these conditions, pipette resistances were about 12 MΩ.

The pipette solution used for chloride current recordings was composed of 30 mM CsCl, 2 mM MgCl_2_, 1 mM CaCl_2 _(free Ca^2+ ^about 50 nM), 5 mM EGTA, 2 mM MgATP, 10 mM Hepes/Tris (pH 7.2) and 410 mM D-mannitol. The bath solution contained 50 mM CsCl, 15 mM CaCl_2_, 2 mM MgCl_2_, 10 mM Mes/Tris (pH 5.7) and 345 mM D-mannitol. In these conditions, patch pipettes had resistances of about 15 MΩ.

## Competing interests

The author(s) declare that they have no competing interests.

## Authors' contributions

EH carried out the patch-clamp recordings, except those regarding endogenous anion currents, participated in the protoplast transformation and drafted the manuscript. GD designed and obtained the expression vectors used in this work, managed the protoplast transformation, performed the confocal microscopy and drafted the manuscript. AAV performed the patch-clamp recordings of the endogenous anion currents and cell wall detection experiments. AC participated in the protoplast transformation and obtainig of expression vectors. HS participated in the design and the coordination of the study and helped to draft the manuscript. JBT participated in the design and the coordination of the study and managed the writing of the manuscript.

## Supplementary Material

Additional File 1 **Supplemental information on the vectors and on the cloning procedure used in this study**. This 2 page file (pdf format) displays 2 figures (and legends) showing (i) a schematic representation of the vectors used in this work and (ii) the procedure for cloning cDNAs in these vectors. Click here for file
