# Supplementary material for: A procedure for localisation and electrophysiological characterisation of ion channels heterologously expressed in a plant context
Source: Plant Methods. 2005 Dec 19;1:14. doi: 10.1186/1746-4811-1-14 (PMC1352354; doi:10.1186/1746-4811-1-14)
Supplement: Additional File 1 — Supplemental information on the vectors and on the cloning procedure used in this study. This 2 page file (pdf format) displays 2 figures (and legends) showing (i) a schematic representation of the vectors used in this work and (ii) the procedure for cloning cDNAs in these vectors. [file 1746-4811-1-14-S1.pdf]

Supplementary material

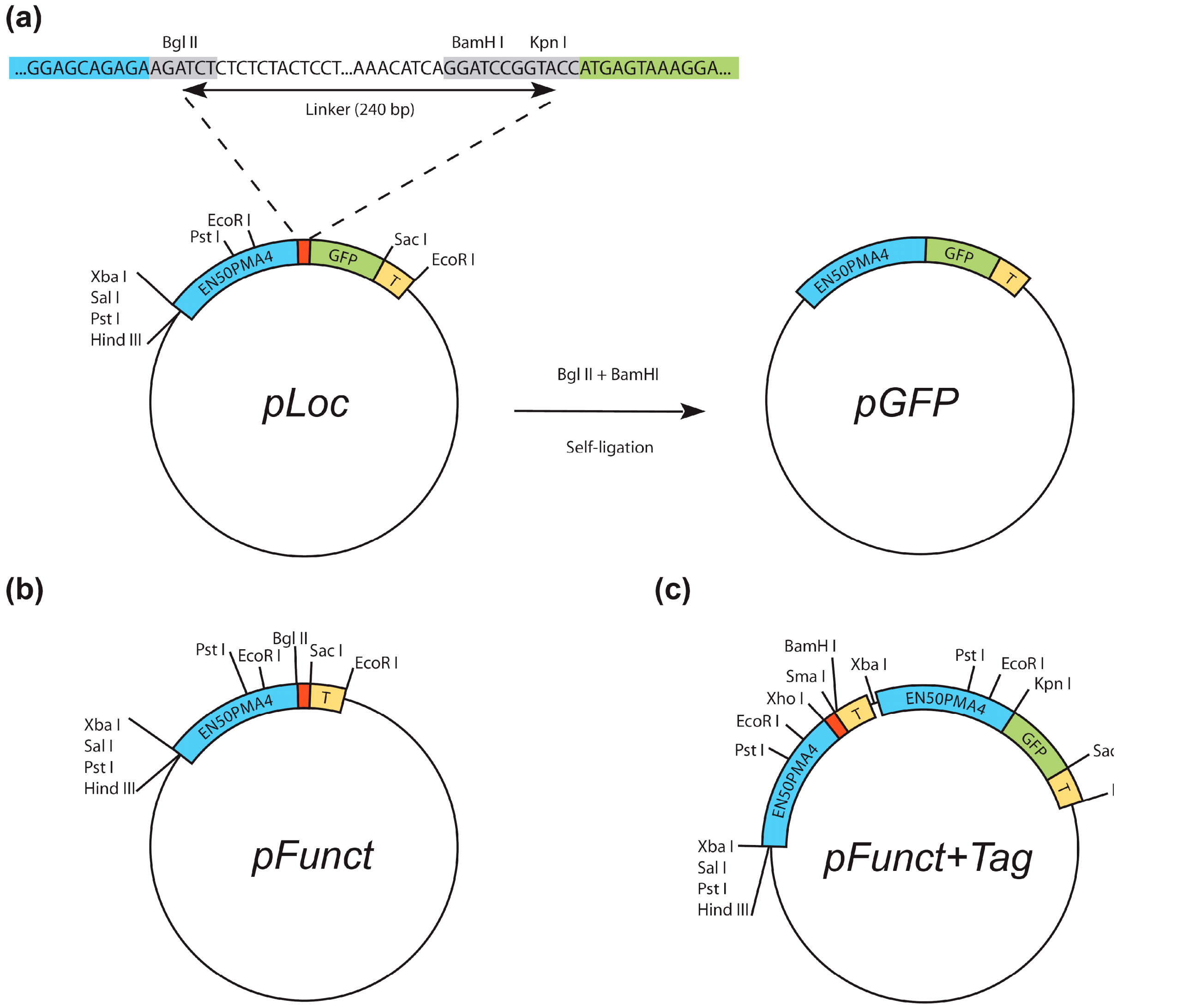

Figure 1. Schematic presentation of the plasmids used for the transient expression in Tobacco protoplasts of a protein alone, or in fusion with the GFP, or in co-expression with the GFP. (a) *pLoc* and *pGFP* plasmids used for the expression of a protein with the GFP fused at its C-terminus or of the GFP alone, respectively. For the *pLoc* plasmid, the nucleotide sequences surrounding the restriction sites at the sides of the linker region are indicated for in-frame cloning of the protein. The *pGFP* plasmid results from the *pLoc* plasmid after removal of the *pLoc* linker with a Bgl II – BamH I enzymatic restriction. (b, c) *pFunct* and *pFunct+Tag* plasmids used for the functional expression of a protein alone or, for cell fluo-tagging purpose, in tandem with the GFP, respectively. The main restriction sites are indicated. *EN50PMA4*: modified *PMA4* promoter (Zhao et al., 1999, *Plant Sci.* **149,** 157), 2509 bp; T: nopaline synthase terminator, 267 bp; GFP: enhanced green fluorescent protein; 723 bp. The background plasmid is the *pTZ-19U* plasmid (Stratagene, LaJolla, CA, USA).

Supplementary material (continued)

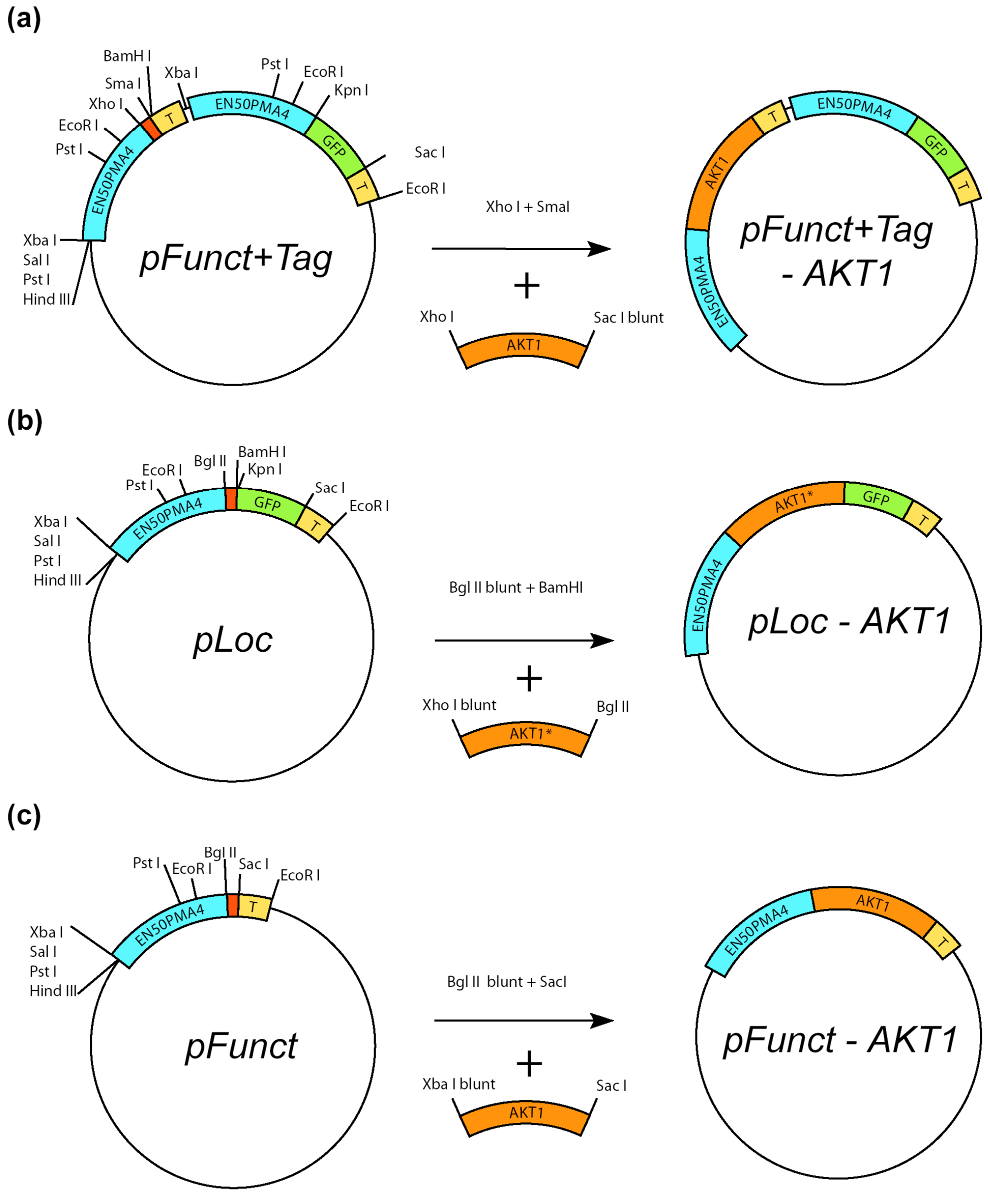

Figure 2. Cloning of *AKT1* and *KAT1* cDNAs in the *pFunct+Tag, pLoc and pFunct* vectors. Only the procedures for AKT1 are represented. (a) Obtaining of the *pFunct+Tag – AKT1* plasmid. The *AKT1* cDNA bordered with XhoI and blunt SacI sites was cloned in the *pFunct+Tag* plasmid opened with XhoI – SmaI endonucleases. (b) Obtaining of the *pTag – AKT1* plasmid. The *AKT1* cDNA deprived by PCR of its stop codon and bordered with blunt XhoI and BglII restriction sites was cloned in frame with the GFP in the *pLoc* plasmid opened with a blunt BglII and BamHI enzymatic restriction. (c) Obtaining of the *pFunct – AKT1* plasmid. An *AKT1* cDNA fragment bordered by blunt XbaI and SacI sites was cloned in the *pFunct* plasmid opened with a blunt BglII and SacI enzymatic restriction. Cloning KAT1 cDNA in the *pFunct+Tag*, *pLoc* and *pFunct* vectors used the same restriction sites and endonucleases. The *pFunct – KAT1* recombinant vector was obtained by integrating the *KAT1* cDNA bordered with the BamH I and Sac I restriction sites in the *pFunct* plasmid opened with a Bgl II and Sac I restriction. The main restriction sites are indicated. EN50PMA4: modified PMA4 promoter (Zhao *et al.*, 1999, *Plant Sci.* **149,** 157), 2509 bp; T: nopaline synthase terminator, 267 bp; GFP: enhanced green fluorescent protein; 723 bp; AKT1: AKT1 cDNA, 2705 bp; AKT1*: AKT1 cDNA deprived of its stop codon.
